# Gut Microbiome Mediates the Causal Link Between Autism Spectrum Disorder and Dietary Preferences: A Mendelian Randomization Study

**DOI:** 10.3390/ijms27042006

**Published:** 2026-02-20

**Authors:** Yuqi Wu, Oscar W. H. Wong, Sizhe Chen, Yun Wang, Guoqing Zhang, Ying Gao, Francis K. L. Chan, Siew Chien Ng, Qi Su

**Affiliations:** 1 Microbiota I-Center (MagIC), Hong Kong SAR 999077, China; 2 Department of Medicine and Therapeutics, The Chinese University of Hong Kong, Hong Kong SAR 999077, China; 3 Department of Psychiatry, The Chinese University of Hong Kong, Hong Kong SAR 999077, China; 4 The D.H. Chen Foundation Hub of Advanced Technology for Child Health (HATCH), The Chinese University of Hong Kong, Hong Kong SAR 999077, China; 5 Shanghai Institute of Nutrition and Health, Chinese Academy of Sciences, Shanghai 200000, China; 6 Centre for Gut Microbiota Research, The Chinese University of Hong Kong, Hong Kong SAR 999077, China; 7 Li Ka Shing Institute of Health Sciences, State Key Laboratory of Digestive Disease, Institute of Digestive Disease, The Chinese University of Hong Kong, Hong Kong SAR 999077, China; 8 New Cornerstone Science Laboratory, The Chinese University of Hong Kong, Hong Kong SAR 999077, China

**Keywords:** autism spectrum disorder, dietary preferences, gut microbiome, mediation effects

## Abstract

Autism spectrum disorder (ASD) frequently co-occurs with malnutrition and gut dysbiosis, yet the underlying mechanisms remain poorly understood. Herein, this cross-sectional study first profiles dietary intake differences using dietary records from 210,874 participants (ASD = 232; non-ASD = 210,642; median age = 56.18) from the UK Biobank (UKB). Second, a bi-directional Mendelian Randomization (MR) approach serves to dissect relationships between ASD genetic susceptibility and dietary preferences by leveraging genome-wide association metadata from the iPSYCH-PGC (ASD) and UKB (dietary intake/food-liking traits). The same strategy is implemented to identify ASD-associated gut microbial species. Mediation analyses further assess the role of gut microbiota in the association between ASD and dietary preferences. Subjects with ASD exhibit higher consumption of cheese, processed meat, and oily fish, alongside lower intake of fruits, and demonstrate a preference for high-fat/salt and energy-dense foods. Additionally, the depletion of *Turicibacter*, *Streptococcus*, and *Lachnospiraceae NK4A136* was causally related with ASD (all false discovery rate < 0.05; *β* = −0.15, *β* = −0.10, *β* = −0.093, respectively), which significantly mediates the ASD-associated elevated preference for high-fat/salt foods. In conclusion, ASD is associated with specific dietary preferences, likely mediated via gut microbiota, highlighting the future potential of gut microbiome-based therapeutics to modify eating disorders for ASD.

## 1. Introduction

Autism spectrum disorder (ASD) is a common neurodevelopmental disorder, commonly accompanied by eating disorders [1,2,3]. The high prevalence of disordered eating behaviors—such as selective/picky eating and binge eating—in individuals with ASD often complicates nutritional management in this vulnerable population [1,3]. This not only influences food preferences but also manifests in altered food intake patterns (i.e., actual consumption frequency and variety), ultimately contributing to nutritional imbalance [4,5]. While some studies attributed the dietary preference in ASD to altered brain patterns in sensory and executive functions, the underlying mechanism is still unclear [6]. There are some mice experiments that have shown that the gut microbiome can influence host dietary choices [7,8]. Given the established links between ASD and the gut microbiome via the gut–brain axis (GBA), the gut microbiome may have multifaceted effects on shaping dietary preferences [9,10,11]. Current evidence, however, remains constrained by small sample sizes and the lack of longitudinal cohorts, which limit causal inference [12]. Furthermore, the mechanisms by which the gut microbiome may differentially influence dietary behavior across ASD subgroups are poorly understood [12,13].

In traditional epidemiological research, observational studies are limited to detecting associations and are prone to confounding and reverse causation, while Mendelian Randomization (MR) bridges this inferential gap by leveraging genetic variants as instrumental variables. This approach mimics the design of a randomized controlled trial, and reduces common biases, offering more robust evidence for causal inference between exposures and outcomes.

Previous studies have demonstrated significant correlations between dietary preferences and heritability [14,15], as well as the genetic determinants of specific eating habits [15], highlighting the complex interplay between genetics and nutrition in shaping individual food choices. Given the strong associations between genetic factors and ASD and gut microbiota [16], we hypothesize that genetic predisposition to ASD, individual dietary preferences, and the gut microbiome form a tightly interconnected triad, with the microbiome serving as a key mediator in these interactions. This integrative framework moves beyond prior isolated associations to explicitly model the microbiome as a functional bridge between ASD and diet.

In this study, we first conducted a cross-sectional analysis to investigate the associations between ASD and dietary preferences using the UK Biobank dataset. We then performed bi-directional MR to rule out the possibility of reverse causality. Finally, recent genome-wide association (GWAS) data on gut microbial taxa were utilized to explore the mediating roles of specific bacterial species in relation to autism-related dietary preferences for unhealthy foods. These findings underscore the potential of targeting gut microbiota as an intervention strategy to address dietary disorders in individuals with ASD.

## 2. Results

### 2.1. Dietary Preferences and Quality Score Among Individuals with and Without ASD

A total of 210,874 participants (ASD = 232; non-ASD = 210,642; median age = 56.18, interquartile range [IQR] = 50~63; 44.91% males) from the UK Biobank were included in our analysis. In addition to poorer lifestyle, including higher alcohol and cigarette consumption, individuals with ASD exhibited more frequent intake of processed meats (*p* < 0.001) and oily fish (*p* = 0.002), but lower intake frequencies for fruit (*p* = 0.038) and vegetables (*p* = 0.052) compared to the non-ASD group (Table 1). Although the association with vegetable intake was only marginally significant in the unadjusted model, its significance substantially increased after controlling for age, gender, and BMI. We further found significantly lower average intakes of fiber (mean difference [MD] = −1.29, standard deviation [SD] = 6.81 g/day), calcium (MD = −52.98, SD = 289.75 mg/day), and poultry (MD = −6.45, SD = 17.77 g/day) in the ASD group, and higher intake of cheese (MD = 0.30, SD = 0.35 slice/day). The results maintained congruent patterns in the sensitivity analysis where age, gender, and BMI were matched to account for sample imbalance (Supplementary Table S1).

### 2.2. Atypical Dietary Intakes Were Associated with Genetic Predisposition in ASD

**Figure 1:**
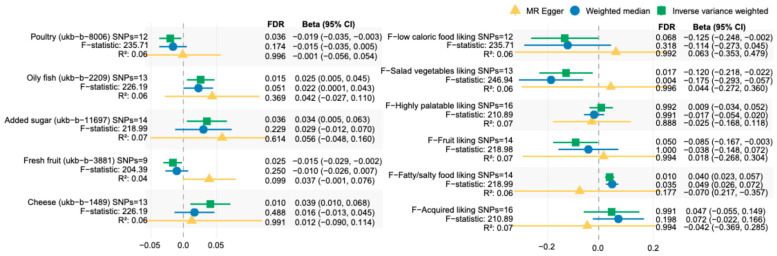
Two-sample Mendelian Randomization estimates for the effect of autism spectrum disorder (ASD) on dietary preferences. nSNP, the number of SNPs used as instrumental variables, with their mean F-statistics and total R^2^ shown below; FDR, false discovery rate; IVW, inverse-variance weighted method. The left panel represents dietary consumption of individual foods and an overall healthy dietary pattern, while the right panel represents food-liking traits based on the algorithmized categories. The FDR was corrected instead of using *p* values through the Benjamini–Hochberg method.

To measure the impact of genetic factors of ASD on dietary intake, 16 instrumental variables (IVs) previously identified as genetic predisposition to ASD were harmonized with outcome traits for MR analysis. Results indicated that ASD condition was associated with a 0.034-times higher frequency of added sugar consumption (IVW: β = 0.034, 95% interval confidence [CI] = 0.005, 0.063, false discovery rate [FDR] = 0.036), along with elevated frequent intake of oily fish (IVW: β [95%CI] = 0.025 [0.005, 0.045], FDR = 0.004) and cheese per week (IVW: β [95%CI] = 0.039 [0.010, 0.068], FDR = 0.010) (Figure 1). Genetically predicted ASD, conversely, showed associations with 0.019 and 0.015 lower weekly frequencies for consuming poultry (IVW: β [95%CI] = −0.019 [−0.035, −0.003], FDR = 0.036) and fresh fruit (IVW: *β* [95%CI] = −0.015 [−0.029, −0.002], FDR = 0.025), respectively (Figure 1; Supplementary Table S2). These associations were robust to heterogeneity and pleiotropy, with leave-one-out sensitivity analysis showing no substantial changes (Figure S2) and null causation in reverse MR (Supplementary Tables S2 and S3). Additionally, algorithm-based categories of food-liking traits revealed similar results, including a decreased liking for salad vegetables (IVW: β [95%CI] = −0.120 [−0.218, −0.022]), fruit (IVW: β [95%CI] = −0.085 [−0.167, −0.003]), and low-caloric food (IVW: β [95%CI] = −0.125 [−0.248, −0.002]), but an enhanced preference for fatty/salty foods (IVW: β [95%CI] = 0.040 [0.023, 0.057]) in ASD individuals (Figure 1 and Supplementary Table S2).

We also included appetite-related hormones and dietary preference-related biomarkers in our analysis, and the results demonstrated significant associations between genetic determinants of ASD and elevated levels of fibroblast growth factor (FGF)-21 (IVW: *β* [95%CI] = 0.425 [0.051, 0.799]), FDR = 0.029), and decreased GLP-1 stimulated insulin secretion with the effect size of −3.010 [−5.380, −0.640] (FDR = 0.030) in the MR-Egger result (Supplementary Tables S2 and S3). These findings suggest that individuals with ASD may have altered metabolic responses that influence their dietary preferences and appetite regulation.

### 2.3. Bi-Directional Interactions Between Gut Microbiota and the Genetic Factors of ASD

**Figure 2:**
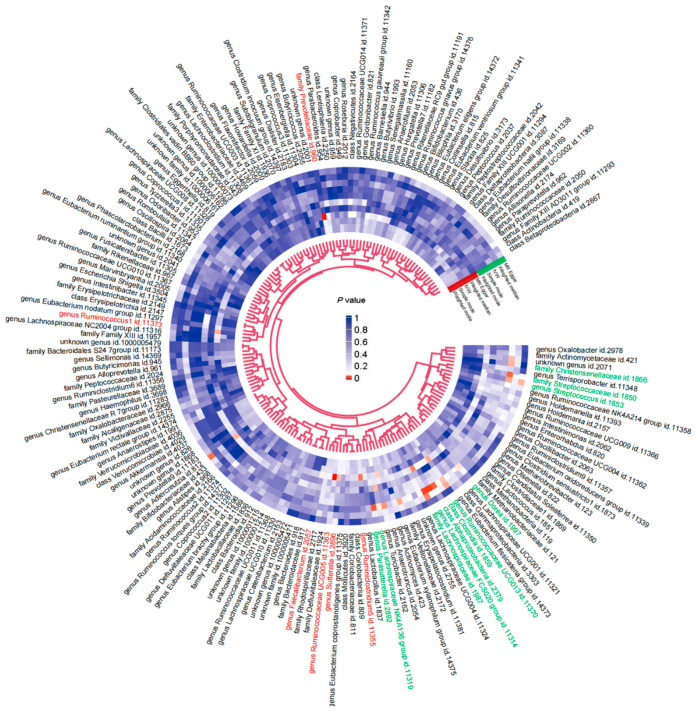
Bi-directional Mendelian Randomization (MR) evaluation of the relationships between autism spectrum disorder (ASD) and the gut microbiome. Estimates of genetic predisposition to ASD on gut microbial taxa (outer circle) and the reverse associations (inner circle). The *p* values from five MR approaches (MR-Egger, weighted median, inverse-variance weighted [IVW], simple mode, and weighted mode) are presented, with the MR-Egger results shown outermost, followed by the other methods moving inward. Specific microbial features with statistically significant IVW estimates were highlighted in red (gut microbiome impacts on ASD) and green (ASD impacts on gut microbiome).

Considering the relationship between the gut microbiome in ASD development as suggested [17], we first tested the impact of gut microbiome-related genetic factors on ASD in the MR analysis. Consistent with previous works [18,19], our results revealed an enrichment of *Faecalibacterium*, while a depletion of four genera, including *Ruminococcus*, *Ruminiclostridum*, *Ruminococcaceae*, and *Sutterella*, were associated with an increased risk of ASD (Figure 2 and Supplementary Table S4), suggesting the potential role of the gut microbiome in the pathogenesis of ASD.

Conversely, we also found that ASD may lead to reduced abundances of two microbial families (*Lachnospiraceae* and *Streptococcaceae*), two classes (*Alphaproteobacteria* and *Clostridia*), and eight genera (*Dorea*, *Eubacterium xylanophilum*, *Lachnospiraceae FCS020*, *Lachnospiraceae NK4A136*, *Parasutterella*, *Ruminococcaceae UCG013*, *Streptococcus*, and *Turicibacter*), but increased abundance of the *Christensenellaceae* family (IVW: *β* = 0.183, FDR = 0.029) (Figure 2 and Supplementary Tables S5 and S6), with no evidence of reverse causation of these taxa. These findings indicate that genetic factors associated with ASD may play a crucial role in shaping the gut microbiome composition, potentially influencing metabolic pathways and host behaviors.

### 2.4. Gut Microbiome Associates with Dietary Preferences in ASD

To determine potential mediators within the gut microbiome, candidate microbial taxa demonstrating significant associations with genetic determinants of ASD were further treated as exposures, while dietary traits served as outcomes in subsequent analysis. The genera *Lachnospiraceae NK4A136* and *Turicibacter* exhibited the highest number of significant associations with diet. Specifically, a higher abundance of these genera was linked to increased consumption of cheese and oily fish while also being associated with decreased salad intake (Supplementary Tables S7 and S8).

**Figure 3:**
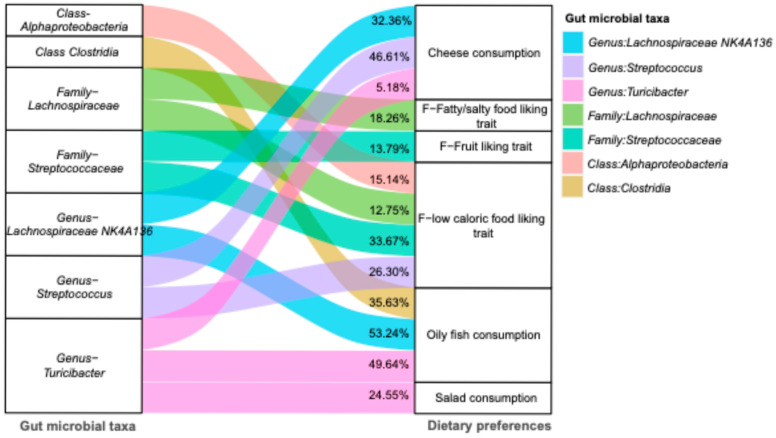
Mediation analysis identified specific gut microbial taxa mediating the associations between genetic predisposition to autism spectrum disorder (ASD) and dietary preferences. The Sankey plot visualizes the identified gut microbial taxa with significant mediation effects (*p* < 0.05). The pathways from ASD to decreased liking of fruits and low-caloric food, which were mediated by the family *Streptococcaceae* and family *Lachnospiraceae*, exhibited only marginal statistical significance. The colors correspond to the linkages mediated by specific gut microbial taxa, with the mediated proportion shown on the top layer.

In the mediation analysis, three bacterial genera—*Turicibacter*, *Streptococcus*, and *Lachnospiraceae NK4A136*—were found to be depleted in individuals with ASD and demonstrated significant mediating roles between ASD and dietary preferences of high-fat or energy-dense foods (Figure 3 and Supplementary Table S9). The effect sizes and contributions of individual microbiome-related SNPs were evaluated (Supplementary Table S10), with rs140912403 and rs992074 accounting for the largest proportion (>30%) of the mediated effects of low-calorie and fatty/salty food-liking traits, respectively. Additionally, the *Lachnospiraceae* family was identified as a mediator between ASD and the preference for fatty/salty foods, accounting for 18.26% of the total effect. Furthermore, *Turicibacter* also served as a mediator, explaining 24.55% of the association between ASD and reduced salad consumption.

### 2.5. Estimates of Genetic Functional Connections Between Gut Microbiome and Dietary Preferences in ASD

**Figure 4:**
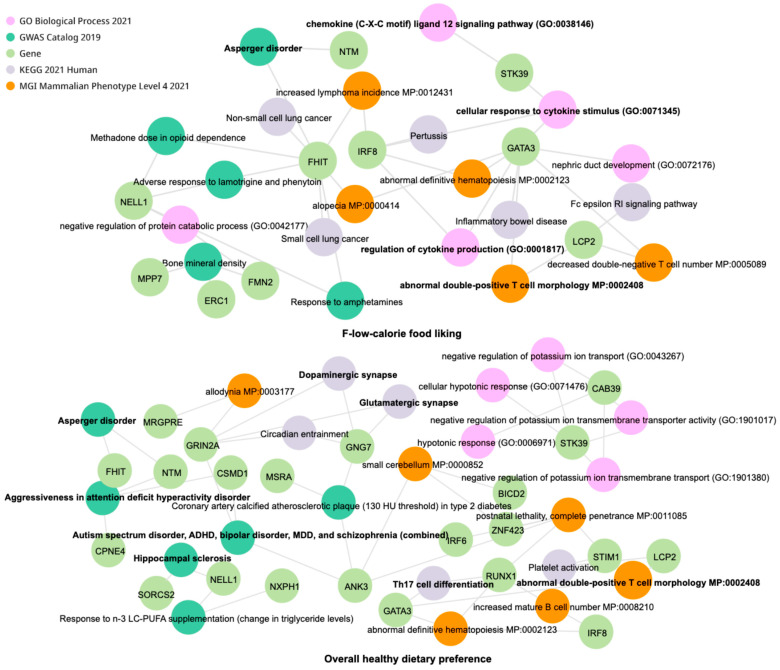
Gene Ontology (GO) biology and disease network enrichment analysis. The overlaps and differences in the enrichment pathways of microbial taxa-associated genetic variants that were significantly associated with the trait of F-low-calorie food-liking (4 gut microbial taxa) and overall healthy diet (8 gut microbial taxa) were analyzed.

Considering the potential influence of host genetic regulation on gut microbial function and its connection to dietary preferences, we further estimated biological signals of those significant genetic variants to provide a hint linking with the pathogenesis of ASD. The GO biology and disease network analysis utilized host genes significantly associated with four gut microbial taxa linked to low-calorie food-liking, as well as eight taxa linked to overall healthy dietary preference. Intriguingly, Asperger’s disorder and immune dysfunction between two networks overlapped (Figure 4). Network enrichment analysis of overall healthy dietary preference revealed significant enrichment in KEGG of the dopaminergic synapse, glutamatergic synapse, and Th17 cell differentiation, suggesting a potential mechanistic link among ASD, the gut microbiome, and dietary preferences. Beyond phenotypic enrichment of the abnormal double-positive T cell morphology (MP:0002408) and chemokine/cytokine-involved GO pathways (GO:0038146, GO:0071345, GO:0001817), the gutMGene database [20] revealed that the ASD-depleted taxa, including *Turicibacter*, *Lachnospiraceae NK4A136*, and *Streptococcus*, specifically suppress pro-inflammatory signals, such as IL-1beta, IL-6, CCL20, and CXCL6. These cytokines may further induce neuroinflammation in the hypothalamic feeding centers and dopaminergic reward processing, thereby driving abnormal dietary preferences.

## 3. Discussion

In this study, consistent with observational findings, there was an enhanced preference for high-fat foods and a reduced liking for fruit/vegetables influenced by genetic determinants of ASD. The gut microbiome exhibited bi-directional roles in ASD. Among the 13 microbial taxa genetically associated with ASD, *Clostridia*, *Turicibacter*, *Lachnospiraceae*, and *Streptococcus* were further explored as significant mediators of the associations between genetic predisposition to ASD and less healthy dietary preferences. These findings provide new insights into leveraging these microbial taxa to improve healthy dietary patterns and nutritional status in the context of ASD.

Although previous research suggests the crucial role of the gut microbiome in the pathogenesis of ASD [17], our bi-directional MR studies revealed that ASD may also contribute to a specific microbial profile, such as the depletion of *Lachnospiraceae*, *Turicibacter*, and *Streptococcus*, serving as potential biomarkers for ASD diagnosis [16]. Moreover, certain microbial taxa can influence appetite and food preferences via the GBA, potentially impacting feeding behaviors through pathways like the hypothalamus-mediated integration of hormonal, sensory, and nutritional cues [1,21].

One of the key findings was the depletion of *Streptococcus*, *Lachnospiraceae NK4A136*, and *Turicibacter* in ASD individuals, which was strongly associated with increased consumption of high-fat foods. This is likely mediated by microbial metabolites like SCFAs, ClpB (a bacterial protein mimetic of α-MSH) and bile acids (BAs), which can impact feeding behavior through c-fiber vagal sensory afferent circuits and hypothalamic regulation [9,22]. Specifically, SCFAs and downstream metabolites derived from *Streptococcus* were involved in regulating eating behaviors via the GBA, showing potential for reducing high-calorie food preferences and lower subjective hunger ratings [23]. Meanwhile, the pronounced proteolytic capacity of *Streptococcus*—which is enriched in hosts on high-protein diets [24]—together with the positive correlation between host L-leucic acid and Streptococcus overabundance, suggested that Streptococcus-derived metabolites may stimulate host reward pathways [25,26], thereby reinforcing a preference for dairy products. Moreover, *Lachnospiraceae NK4A136*, another key SCFA producer, has also been found to be positively associated with adherence to a Mediterranean diet [27], possibly due to its effects on appetite regulation through leptin and PYY [28]. Besides the role of SCFAs, BAs which are predominantly metabolized by *Turicibacter* trains [29] can regulate the secretion of GLP-1 and PYY via BA receptors, leading to a decrease in overconsumption [30]. In line with our findings, *Turicibacter* was consistently depleted in ASD regardless of dietary shift [31,32], suggesting its depletion should be upstream of dietary intake rather than the consequence of diet. Nevertheless, horizontal pleiotropy, where ASD-related genetic variants independently influence the microbiome and dietary preferences—cannot be entirely excluded and requires further exploration.

In addition to the microbial metabolites, certain hormonal alterations observed in individuals with ASD—such as elevated FGF-21—may further interact with the gut microbiome [33], potentially driving a shift in dietary preference towards high salt [34] or fatty/protein-rich savory foods [35,36]. Nevertheless, given the multifaceted role of FGF-21 as a distinct endocrine regulator in dietary control, its mechanistic impact on sweet taste perception and preference—particularly within the context of carbohydrate and energy balance—warrants cautious interpretation, considering a state of FGF-21 resistance or a broader metabolic stress response [37,38]. Furthermore, the gene enrichment analysis linked the identified gut microbes to immune dysfunction pathways in ASD, such as TLR4-mediated modulation of appetite-regulating neuropeptides [39]. These cues require us to further explore how gut microbiota products regulate dietary preferences through immune responses in ASD.

The largest sample size with well-qualified GWAS data allowed the identification of specific microbial taxa mediating the associations between genetic predisposition to ASD and dietary preferences. However, several research limitations should be noted. First, given the distinct patterns of neuroanatomical alterations in ASD [40], the underlying morphological mechanisms linking ASD to dietary preferences warrant further investigation. Second, following the precedent set by previous MR studies [28,41,42], we adopted a more lenient *p* value threshold for selecting genetic instruments for the gut microbiota. This choice was made to balance statistical power with analytical validity; however, it requires a cautious interpretation of results. To address potential concerns, F-statistics for the SNPs were calculated (detailed in Supplementary Tables S2 and S5), which generally demonstrated robust instrument strength. Moreover, despite using robust MR estimators, unmeasured pleiotropy cannot be entirely ruled out. Detailed assessments are provided in Supplementary Table S2, and these causal pathways should be interpreted with caution. Third, there is a conceptual distinction between observed dietary intake and preference as food-liking traits. Third, we acknowledge the absence of food preference measures in our cross-sectional analysis, which may limit the interpretation of MR findings regarding ASD-linked dietary intake and liking traits. However, since dietary preference is a recognized determinant of habitual consumption, the close alignment between our observational intake results and preference-based MR outputs provides converging evidence that these related dietary dimensions consistently associate with ASD and the gut microbiome. Finally, despite the large scale of the UK Biobank, the limited size of the ASD cohort presents a risk of selection bias and the reliance on European-ancestry GWAS may not fully represent broader populations. Although we conducted a matched sensitivity analysis, which yielded congruent and even more robust results, these associations should be validated in diverse populations and to delve deeper into the gut microbiome-mediated mechanism.

In conclusion, our study provides novel insight into the direction of diagnosed ASD and certain undesired dietary preferences in adulthood, highlighting the mediation effects of the gut microbiome. Extensive research avenues will enhance the translational value of the findings, leading to the development of microbiome-targeted interventions for dietary management and healthfulness in individuals with ASD.

## 4. Materials and Methods

### 4.1. Study Design

The UK Biobank is a large-scale, ongoing prospective cohort study that enrolled over 500,000 participants aged 40–69 years between 2006 and 2010. To ensure diverse geographical distribution, participants were recruited from 22 assessment centers spanning England, Wales, and Scotland. The UK Biobank, with an REC reference approval from the research ethics committee (11/NW/0382), integrated longitudinal socio-economic data, biological assays, genomic information, and various health-related outcomes, allowing us to conduct this analysis. Eligible and consenting individuals attended a baseline visit, where they provided written informed consent and completed a touch-screen questionnaire, including the assessment of dietary intake and medical history records.

In this cross-sectional design, we included ASD cases identified through diagnostic summary data recorded with the ICD-10 codes F84.0 (childhood autism), F84.1 (atypical autism), and F84.5 (Asperger’s syndrome), which align with the dimensional diagnostic approach adopted in the Diagnostic and Statistical Manual of Mental Disorders, Fifth Edition (DSM–5) classification of ASD. Descriptions of autism reported in the “Mental health conditions ever diagnosed by a professional” field (Data-field ID: 29000) were also included in the ASD group, while individuals without reported ASD conditions were classified as healthy non-ASD controls. To characterize their dietary profiles, we excluded individuals with incomplete data on basic demographic information, the food frequency questionnaire (FFQ), or 24 h dietary recalls, resulting in a final sample of 210,874 participants for the study.

To conduct a valid MR analysis, three core assumptions should be met: (1) The selected genetic IVs are strongly correlated with the exposure of interest. (2) The IVs are not associated with any confounding factors that could influence the relationship between exposure and outcome. (3) The IVs are not directly related to the outcome, except through their effect on exposure. The two-sample MR study design employed in this analysis is depicted in Supplementary Figure S1A. The ASD GWAS dataset (ID: ieu-a-1185) was sourced from the iPSYCH consortium—an independent study that does not include UK Biobank participants with dietary data, allowing for an investigation of the associations between them with genetic predisposition.

### 4.2. Demographic Characteristics

Socio-economic factors, including education, the Townsend deprivation index (TDI), household income, and employment status, are all recorded in the fields of the UK Biobank. Education attainment was categorized into six ordered levels, while household income was coded as an ordinal variable with five levels. The TDI is a metric used to assess deprivation at the area level, with higher scores indicating greater levels of deprivation. Participants self-reported their employment status, with five classifications (employed, retired, unavailable to work, household caregiving, and unemployed). Health-related factors like smoking status, alcohol consumption, and supplement use were self-reported and available in the UK Biobank as well. More details about the dataset structure can be found elsewhere [21,33].

### 4.3. Dietary Assessment

The UK Biobank dataset includes information from the touch-screen food frequency questionnaire in Category 100052, which contains data on the reported frequency of intake for a range of common food items over the past year, including 0, <1, 1, 2 to 4, 5 to 6, ≥7 times a week. This allows us to assess individuals’ preferences for the frequency of consuming processed meat, oily fish, fruits, and vegetables [43]. Additionally, the 24 h dietary records in Category 100,090 offer information on estimated total energy and daily nutrient intakes, including the amounts of macronutrients, total fiber, and calcium [44]. Unlike habitual dietary intake, which reflects long-term individual preferences, 24 h dietary records of yesterday’s consumption add a complementary dimension to dietary assessment. The Oxford WebQ online 24 h dietary assessment tool—previously validated by urine biomarkers—demonstrates strong consistency with the touch-screen questionnaire. The Healthy Diet Index (HDI) is a food-based and nutrient-based index designed to reflect adherence to the World Health Organization’s (WHO) recommendations for a healthy diet. We adopted an 11-item score, with a higher score indicating a healthier diet [44]. The detailed cut-off criteria for scoring are shown in Supplementary Table S11.

### 4.4. Instrumental Variables in the MR

For the main exposure, the GWAS of ASD was derived from a meta-analysis of the Psychiatric Genomics Consortium (PGC) cohort and the Danish Lundbeck Foundation Initiative for Integrative Psychiatric Research (iPSYCH) cohort, including 18,382 cases and 27,969 controls [45]. The Autism Diagnostic Interview Revised (ADI-R) and/or Autism Diagnostic Observation Schedule (ADOS) were used as diagnostic tools. Data were available from https://gwas.mrcieu.ac.uk/datasets/ieu-a-1185/ (accessed on 28 August 2024). All the data sources have been stated in Supplementary Table S12. ASD-associated SNPs at a genome-wide significance threshold with *p* < 1.0 × 10^−6^ were selected. To minimize deviation stemming from weak IVs, we assessed the F-statistic using the formula F = R^2^ × (n − k − 1)/K × (1 − R^2^), where n is the sample size in the GWAS study, k is the number of IVs, and R^2^ is the proportion of exposure variance explained by the IVs [46]. The mean F-statistic and total R^2^ for the ASD-related IVs were provided in Supplementary Table S2. Exposure and outcome SNPs with an F-statistic > 10 and independent of each other (linkage disequilibrium score regression [LD] r^2^ < 0.001 within 10,000 kb) were selected to mitigate potential bias [46]. Subsequently, SNP effect sizes were harmonized across studies, with exclusion of palindromic and incompatible variants (Supplementary Table S13) [47].

Considering instrument bias and preventing potential sample overlap between exposure and outcome in the two-sample MR analysis, the GWAS summary statistics for the outcome of dietary preferences stemmed from the UK Biobank. We additionally included a UK Biobank GWAS that identified 139 food-/beverage-liking traits and their hierarchical genetic architecture (https://www.ebi.ac.uk/gwas/studies/, accessed on 28 August 2024) [15]. All the food preference traits were determined by a 9-point scale, where the rating scale ranged from 1 (Extreme dislike) to 9 (Extreme like) (https://biobank.ndph.ox.ac.uk/showcase/showcase/docs/foodpref.pdf, accessed on 28 August 2024). A multi-level hierarchical map was constructed to present the liking traits for specific food groups based on the similarity of genetic correlation coefficients with the prefix “F-” [15]. Finally, the map revealed three primary dimensions: “Low-caloric”, “Highly palatable”, and “Acquired” food. The “Highly palatable” dimension, recognized as the preference for highly rewarding foods such as sweets, meat, and fried food, showed a distinct direction with other lower-caloric and stronger-taste intensities (the other two dimensions) with no genetic correlation.

Based on previous findings on the effects of gut microbial taxa on appetite [28], we also applied the MR approach to explore the impact of ASD on poor appetite or overeating and anorexia nervosa as categorical traits and on the appetite-related hormones, including Peptide YY (PYY), glucagon-like peptide-1 (GLP-1), and FGF-21, as continuous variables for further study. All spectrometry-based detection results accounted for fasting time and season, with neither showing significant influence [35,48]. For appetite, the GWAS summary data investigating the answer, “not at all, several days, more than half the days, nearly every day”, to the question of the frequency of experiencing poor appetite or excessive eating [Depressive symptoms] were obtained from the UK Biobank (https://gwas.mrcieu.ac.uk/datasets/ukb-e-20511_CSA/, accessed on 28 August 2024). For now, no study has examined the SNPs associated with other disordered eating behaviors, such as binge eating and selective eating, in the UK Biobank. Therefore, we could only obtain the GWAS data for anorexia nervosa as an alternative representation of disordered eating behaviors (https://r8.risteys.finngen.fi/phenocode/R18_ANOREXIA, accessed on 28 August 2024). The PYY and GLP-1 GWAS summary data were derived from two meta-analysis results (GWAS ID in https://gwas.mrcieu.ac.uk/ (accessed on 28 August 2024), prot-c-3727_35_1 and ebi-a-GCST005353), all of which were of European descent. Of note, Gudmundsdottir et al. [49] further applied this GWAS on GLP-1-stimulated insulin secretion to calculate polygenic risk score, which showed a high correlation with glucose-stimulated insulin secretion in a larger population (the MAGIC cohort). These MR results were validated through sensitivity analysis, further suggesting the absence of heterogeneity and horizontal pleiotropy.

The gut bacterial abundance-associated SNPs used as the instruments of mediators were derived from a previous GWAS study in the Biogen consortium, which identified 211 gut microbial taxa (https://gwas.mrcieu.ac.uk/datasets, accessed on 28 August 2024). The meta-dataset covered 18,340 individuals from 24 cohorts and included 122,110 gut microbial taxa-associated SNPs using direct taxonomic classification methods [31]. We used a more relaxed *p* value threshold of less than 1 × 10^−5^ for selecting IVs from the GWAS statistics to ensure reliable results, as they rarely achieved the significance level required for the entire genome (*p* < 10^−8^) [40]. The cut-off values for the F-statistic and LD of each gut microbiome-associated IV were based on the same criteria. The strength and statistical power of the instrumental variables were summarized in Supplementary Tables S2 and S5.

### 4.5. Statistical Analysis

Macronutrient intakes were presented in percentages of total energy intake (% en) based on the nutrient density approach. Other dietary components were presented in their respective units per day. The differences in individual characteristics, including dietary factors, were evaluated using the Mann–Whitney U test for continuous variables and the exact chi-square test for categorical variables, expressed as mean ± standard deviation (SD) and percentages of individuals, respectively. In the sensitivity analysis, we employed a pairwise propensity score matching (PSM) method to conduct comparisons within an age-, gender-, and BMI-matched population with equal sample sizes across the groups.

To confirm the observational results, the inverse-variance weighted (IVW) approach in the two-sample MR was used as the primary analysis method [46], which is based on the assumption that all valid IVs are not associated with any confounders of the exposure–outcome relationship. The IVW technique yields the most accurate, objective, and effective causal estimations, correcting for IV heterogeneity [46]. The weighted median method and MR-Egger regression were used as complementary approaches to the primary IVW analysis, providing additional evidence-supported estimates based on different underlying assumptions [50,51]. To ensure the reliability of the MR results, horizontal pleiotropy and heterogeneity were examined through MR-Egger intercept analysis (*p* > 0.05 indicates no horizontal pleiotropy) and Cochran’s Q test, respectively. Considering the potential chance to increase the overall type I error during multiple comparisons, we implemented the FDR correction using the Benjamini–Hochberg procedure on the MR results. Additionally, bi-directional MR analyses were performed to exclude potential reverse causations.

Understanding the mechanisms by which the gut microbiome potentially mediates the effect of ASD on dietary preferences could provide novel insights into the potential targets of dietary management in ASD. According to the theoretical foundation of mediation analysis, we have first screened a total of 211 taxa to identify the linkages between ASD and gut microbiome compositions. Those microbial taxa with statistical significance after FDR corrections (IVW method < 0.05) proceeded to an examination of their potential associations with dietary preferences, as well as of the mediating role on ASD-related dietary preferences [43]. To identify potential mediators, a second model estimating the effects of mediators on outcomes based on the two-step MR method was conducted.

Those gut microbiota significantly associated with specific dietary preferences in ASD (*p* < 0.05) were considered candidates for subsequent mediation estimates (Supplementary Figure S1B). These potential mediators were then incorporated into the multivariable Mendelian Randomization (MVMR) analyses as the secondary exposure to calculate the direct effect of ASD on dietary preferences [52]. Then the indirect effect can be estimated by subtracting the direct effect from the total effect based on the IVW result. However, if the IVW results for the total effect did not meet the absence of pleiotropy detection, we adopted MR-Egger results with statistical significance as total effects and calculated the indirect effects based on the two-step MR method [52]. A univariable MR model was conducted to obtain the pure and unconfounded effect of ASD on the gut microbiome, multiplying with the effect of the gut microbiome on the outcome. The confidence intervals (CIs) for the mediation effects were estimated through the delta method [53]. Considering the predominantly European ancestry of study participants, we estimated sample overlap proportions using the intercept term from LD score regression (Supplementary Table S12) [54]. An online calculator (https://sb452.shinyapps.io/overlap/, accessed on 10 October 2024) was used to ensure the type 1 error rate due to sample overlap was under 0.05.

The primary MR and reverse MR analyses were performed using the “TwoSampleMR” package, and MVMR results were estimated using the “MVMR” package within the R software (version 4.2.2; R Core Team, Vienna, Austria). Other statistical analyses were performed using SAS software (version 9.4; SAS Institute Inc., Cary, NC, USA) unless stated otherwise. Gene Ontology (GO) biology and disease network analysis for the host genes related to the identified microbial mediators was carried out via Enrichr (https://maayanlab.cloud/enrichr-kg, accessed on 10 October 2025), which integrated information from four libraries: GO Biological Process 2021, GWAS Catalog 2019, MGI Mammalian Phenotype Level 4 2021, and KEGG 2021 Human. The overall healthy dietary preference represented a trait of lower cheese but higher salad/vegetable consumption, as well as a reduced liking for fatty foods but an increased preference for low-calorie foods. GWAS variants were annotated to proximal genes within a 100 kb flanking region of each lead SNP. The corresponding gene labels and IDs for the identified gut microbiome-associated SNPs were provided in Supplementary Table S14.

## Figures and Tables

**Table 1:** Characteristics and dietary preferences between individuals with and without autism spectrum disorder (ASD) in the UK Biobank (*n* = 210,874) ^1^.

	Non-ASD *n* = 210,642	ASD *n* = 232	*p* Value
Age (years), mean ± SD	56.08 ± 7.95	55.22 ± 8.63	0.173
Male, No. (%)	94,565 (44.89)	148 (63.79)	<0.001
BMI (kg/m^2^), mean ± SD	26.96 ± 4.65	28.52 ± 5.70	<0.001
Highest educational level achieved, No. (%)			<0.001
College or university degree	89,622 (42.55)	98 (42.24)	
Vocational qualifications	22,000 (10.44)	23 (9.91)	
Optional national exams at ages 17–18 years	27,456 (13.03)	30 (12.93)	
National exams at age 16 years	52,555 (24.95)	40 (17.24)	
Others	18,044 (8.57)	36 (15.52)	
Townsend deprivation index (TDI), mean ± SD			
Categories of TDI by quintile, No. (%)			<0.001
Q1 (≤−3.96)	44,667 (21.23)	16 (6.90)	
Q2 (−3.96~−2.79)	44,688 (21.24)	31 (13.36)	
Q3 (−2.79~−1.33)	42,815 (20.35)	41 (17.67)	
Q4 (−1.33~1.35)	42,792 (20.34)	60 (25.86)	
Q5 (>1.35)	35,415 (16.83)	84 (36.21)	
Cigarette smoking, No. (%)			<0.001
Never	118,877 (56.44)	104 (44.83)	
Previous	74,764 (35.49)	92 (39.66)	
Current	16,510 (7.84)	34 (14.66)	
Alcohol consumption, No. (%)			<0.001
Never or special occasions only	34,254 (16.26)	71 (30.60)	
1–3 times/month	23,233 (11.03)	27 (11.64)	
1 or 2 times/week	52,392 (24.87)	46 (19.83)	
3 or 4 times/week	52,579 (24.96)	33 (14.22)	
Daily/almost daily	48,080 (22.83)	55 (23.71)	
Frequency of processed meat intake, No. (%)			<0.001
<1/week	88,599 (42.06)	77 (33.19)	
1–2/week	62,344 (29.60)	68 (29.31)	
≥2/week	59,514 (28.25)	85 (36.64)	
Frequency of oily fish intake, No. (%)			0.002
<1/week	91,104 (43.26)	104 (44.83)	
1–2/week	81,504 (38.69)	75 (32.33)	
≥2/week	37,496 (17.80)	52 (22.41)	
Frequency of vegetable intake, No. (%)			0.052
<1/week	33,777 (16.04)	50 (21.55)	
1–2/week	157,981 (75.00)	160 (68.97)	
≥2/week	18,224 (8.65)	20 (8.62)	
Frequency of fruit intake, No. (%)			0.038
<1/week	70,138 (33.30)	92 (39.66)	
1–2/week	103,626 (49.20)	98 (42.24)	
≥2/week	36,702 (17.42)	41 (17.67)	
Total energy (KJ)	3845.85 ± 2380.87	3747.69 ± 2377.79	0.648
Fat (%)	34.36 ± 8.5	32.93 ± 9.79	0.009
Protein (%)	15.88 ± 3.65	15.39 ± 4.45	0.004
Carbohydrate (%)	49.75 ± 8.73	51.68 ± 10.24	0.001
Fiber (g/day)	7.16 ± 4.86	5.87 ± 4.77	<0.001
Calcium (mg/day)	427.46 ± 274.62	374.48 ± 276.56	<0.001
Cheese (slice ≈ 20 g/day)	0.37 ± 0.26	0.67 ± 0.23	<0.001
Poultry (g/day)	171.01 ± 11.88	64.56 ± 13.22	0.002
HDI score	3.53 ± 1.31	3.51 ± 1.33	0.177

^1^ SD, standard deviation; BMI, body mass index; Q1, the first quintile; SFA, saturated fatty acids; PUFA, polyunsaturated fatty acids. The components and scoring method of the Healthy Diet Indicator (HDI) were detailed in the Supplementary Materials. The differences in individual characteristics, including dietary factors, were evaluated using the Mann–Whitney U test for continuous variables and the exact chi-square test for categorical variables, expressed as mean ± SD and percentages of individuals, respectively.

## Data Availability

The data sources that support the findings of this study are available in the Supplementary Materials, which can be accessed at https://api.opengwas.io/ (accessed on 28 August 2024). The codes and remaining data are available at GitHub Repository (https://github.com/wicky27/ASD-diet-and-microbiome-research.git, public accessible on 6 March 2025).

## Supplementary Materials

The following supporting information can be downloaded at: https://www.mdpi.com/article/10.3390/ijms27042006/s1.
