# Supplementary material for: Gut Microbiome Mediates the Causal Link Between Autism Spectrum Disorder and Dietary Preferences: A Mendelian Randomization Study
Source: Int J Mol Sci. 2026 Feb 20;27(4):2006. doi: 10.3390/ijms27042006 (PMC12941152; doi:10.3390/ijms27042006)
Supplement: Supplementary file 1 [file ijms-27-02006-s001.zip › STROBE MR checklist.pdf]

STROBE-MR checklist of recommended items to address in reports of Mendelian randomization studies[1 2]

Item No.	Section	Checklist item	Page No.	Relevant text from manuscript
1	**TITLE and ABSTRACT**	Indicate Mendelian randomization (MR) as the study's design in the title and/or the abstract if that is a main purpose of the study	Page 1	"the cross-sectional study ...,a bi-directional Mendelian Randomization (MR) approach." in abstract.
	**INTRODUCTION**			
2	**Background**	Explain the scientific background and rationale for the reported study. What is the exposure? Is a potential causal relationship between exposure and outcome plausible? Justify why MR is a helpful method to address the study question	Page 2	" Given the established links between ASD and gut microbiome via the gut-brain axis (GBA), the gut microbiome may have multifaceted effects on shaping dietary preferences....In traditional epidemiological research, observational studies are limited to detecting associations and are prone to confounding and reverse causation, while Mendelian Randomization (MR) bridges this inferential gap by leveraging genetic variants as in-strumental variables. This approach mimics the design of a randomized controlled trial, and reduces common biases, offering more robust evidence for causal inference between exposures and outcomes. " justify that MR is a helpful method.
3	**Objectives**	State specific objectives clearly, including pre-specified causal hypotheses (if any). State that MR is a method that, under specific assumptions, intends to estimate causal effects	Page 2	" We then performed bi-directional Mendelian Randomization.to rule out the possibility reverse causality. Finally, recent genome-wide associations (GWAS) data on gut microbial taxa were utilized to explore the mediating roles of specific bacterial species in relation to autism-related dietary preferences for unhealthy foods."
	**METHODS**			
4	**Study design and data sources**	Present key elements of the study design early in the article. Consider including a table listing sources of data for all phases of the study. For each data source contributing to the analysis, describe the following:	Page 11	"For the main exposure, the GWAS of ASD...All the data sources have been stated in the table S9. .."
	a)	Setting: Describe the study design and the underlying population, if possible. Describe the setting, locations, and relevant dates, including periods of recruitment, exposure, follow-up, and data collection, when available.	Page 10	Details have been described in the cited references.<br>"The UK Biobank is a large-scale, ongoing prospective cohort study that enrolled over 500,000 participants aged 40–69 years between 2006 and 2010. To ensure diverse geo-graphical

					distribution, participants were recruited from 22 assessment centers spanning England, Wales, and Scotland. The UK Biobank, with a REC reference approval from the research ethics committee (11/NW/0382), integrated longitudinal socio-economic data, biological assays, genomic information, and various health-related outcomes, allowing us to conduct this analysis. “
		b)	Participants: Give the eligibility criteria, and the sources and methods of selection of participants. Report the sample size, and whether any power or sample size calculations were carried out prior to the main analysis	Page 10-11	“The UK Biobank is a large, ongoing population-based cohort study comprising over 500,000 individuals aged 40-69 years,..To characterize their dietary profiles, we excluded individuals with incomplete data on basic demographic information, food frequency questionnaire (FFQ), or 24-hour dietary recalls, resulting in a final sample of 210,874 participants for the study...The meta-dataset covered 18,340 individuals from 24 cohorts and included 122,110 gut microbial taxa-associated SNPs using..”
		c)	Describe measurement, quality control and selection of genetic variants	Page 11	“ASD-associated SNPs at a genome-wide significance threshold with $P < 1.0\times10^{-6}$ were selected. To minimize deviation stemming from weak IVs, we assessed the F-statistic using the formula: $F= R^2\times (n-k-1)/K\times (1-R^2)$, where n is the sample size in the GWAS study, k is the number of IVs, and R^2 is the proportion of exposure variance explained by the IVs (Burgess et al. 2011). SNPs that be in strong linkage disequilibrium (LD) according to the cutoff ($R^2 < 0.001$, region size = 10,000 kb) or have an F-statistic $\leq$ 10 were excluded to mitigate potential bias (Burgess et al. 2011).”
		d)	For each exposure, outcome, and other relevant variables, describe methods of assessment and diagnostic criteria for diseases	Page 10	“Autism Diagnostic Interview-Revised (ADI-R) and/or Autism Diagnostic Observation Schedule (ADOS) were used as diagnostic tools....”
		e)	Provide details of ethics committee approval and participant informed consent, if relevant	Page 10	in the cited references. (not provide due to word limits) “with a REC reference approval from the research ethics committee (11/NW/0382),..”
5	**Assumptions**		Explicitly state the three core IV assumptions for the main analysis (relevance, independence and exclusion restriction) as well assumptions for any additional or sensitivity analysis	Page 10-11, Supplementary Table S5	“To conduct a valid MR analysis, three core assumptions should be met:...allowing for the investigation of causal relationships between them.”

6	**Statistical methods: main analysis**		Describe statistical methods and statistics used		
		a)	Describe how quantitative variables were handled in the analyses (i.e., scale, units, model)	Page 11	"Additionally, one previous GWAS study evaluated genetic correlations with 139 food- and beverage-liking traits and identified a multi-level hierarchical map...Finally, the map revealed three primary dimensions: .." and "The gut bacterial abundance associated SNPs used as the instruments of mediators..."
		b)	Describe how genetic variants were handled in the analyses and, if applicable, how their weights were selected	Page 11	"ASD-associated SNPs at a genome-wide significance threshold with $P < 1.0\times10^{-6}$ were selected. .... in strong linkage disequilibrium (LD)..."
		c)	Describe the MR estimator (e.g. two-stage least squares, Wald ratio) and related statistics. Detail the included covariates and, in case of two-sample MR, whether the same covariate set was used for adjustment in the two samples	Page 12-13	"To confirm the observational results, the IVW approach in the Two-sample MR was used as the primary analysis method ....The weighted median method and MR-Egger regression were used as complementary approaches to the primary IVW analysis, providing additional evidence-supported estimates based on different underlying assumptions"
		d)	Explain how missing data were addressed		
		e)	If applicable, indicate how multiple testing was addressed	Page 13	"Considering the potential chance to increase the overall type I error during multiple comparisons, we implemented the FDR correction using the Benjamini–Hochberg procedure on the MR results."
7	**Assessment of assumptions**		Describe any methods or prior knowledge used to assess the assumptions or justify their validity	Page 13	Various measures were implemented to confirm the validity of the assumptions. For example, "We used a more relaxed *P* value threshold of less than 1×10^{-5} for selecting IVs from the GWAS statistics to ensure reliable results, as they rarely achieved the significance level required for the entire genome ($P < 10^{-8}$) (Sanna et al. 2019). The cutoff values for the F-statistic and LD of each gut microbiome-associated IVs were based on the same criteria."

8	**Sensitivity analyses and additional analyses**	Describe any sensitivity analyses or additional analyses performed (e.g. comparison of effect estimates from different approaches, independent replication, bias analytic techniques, validation of instruments, simulations)	Page 12	"These MR results were validated through the sensitivity analysis, further suggesting the absence of heterogeneity and horizontal pleiotropy." and "To ensure the reliability of the MR results,...Cochran's Q test, respectively."
9	**Software and pre-registration**			
	a)	Name statistical software and package(s), including version and settings used	Page 13	"The primary MR and reverse MR analyses were performed using the "TwoSampleMR" package, and MVMR results were estimated using the "MVMR" package within the R software (version 4.2.2; R Core Team, Vienna, Austria).."
	b)	State whether the study protocol and details were pre-registered (as well as when and where)	Page 10	in the cited references. (not provide due to word limits, the UKB study was described as "Eligible and consenting individuals attended a baseline visit, where they provided written informed consent and completed a touchscreen questionnaire")
	**RESULTS**			
10	**Descriptive data**			
	a)	Report the numbers of individuals at each stage of included studies and reasons for exclusion. Consider use of a flow diagram	Page 10, 13	" Eligible and consenting individuals attended a baseline visit, where they provided written informed consent and completed a touchscreen questionnaire, including the assessment of dietary intake and medical history records...", "5.4 Instrumental Variables in the MR..", and "In the sensitivity analysis, we employed a pairwise propensity score matching (PSM) method to conduct comparisons within an age-, gender-, and BMI-matched population with equal sample sizes across the groups" in the methods.
	b)	Report summary statistics for phenotypic exposure(s), outcome(s), and other relevant variables (e.g. means, SDs, proportions)	Page 10, 11,Table S5	"To address potential concerns, F-statistics for the SNPs were calculated (detailed in Sup-plementary Tables S2 and S5), which generally demonstrated robust instrument strength. Moreover, despite using robust MR estimators, unmeasured pleiotropy cannot be entirely ruled out..." and "To minimize deviation stemming from

					weak IVs, we assessed the F-statistic using the formula...”
		c)	If the data sources include meta-analyses of previous studies, provide the assessments of heterogeneity across these studies		NA
		d)	For two-sample MR:<br>i. Provide justification of the similarity of the genetic variant-exposure associations between the exposure and outcome samples<br>ii. Provide information on the number of individuals who overlap between the exposure and outcome studies	Page 13	“Considering the predominantly European ancestry of study participants, we estimated sample overlap proportions using the intercept term from LD score regression ...The online calculator (https://sb452.shinyapps.io/overlap/) was used to ensure the type 1 error rate due to sample overlap was under 0.05.”
11	**Main results**				
		a)	Report the associations between genetic variant and exposure, and between genetic variant and outcome, preferably on an interpretable scale	Table S12	Table S12. Valid instrumental variables (IVs) used to estimate the effects of autism spectrum disorder (ASD) on identified dietary preferences and associated traits in the two-sample Mendelian Randomization study.
		b)	Report MR estimates of the relationship between exposure and outcome, and the measures of uncertainty from the MR analysis, on an interpretable scale, such as odds ratio or relative risk per SD difference	Page 4	“To measure the impact of genetic factors of ASD on the dietary intakes,... Results showed that several SNPs were positively associated with elevated intakes for consuming added sugar (Inverse variance weighted [IVW]: β = 0.034,..”
		c)	If relevant, consider translating estimates of relative risk into absolute risk for a meaningful time period		NA
		d)	Consider plots to visualize results (e.g. forest plot, scatterplot of associations between genetic variants and outcome versus between genetic variants and exposure)	Page 5,6	Figure 1 and figure 2
12	**Assessment of assumptions**				
		a)	Report the assessment of the validity of the assumptions	Page 11	“The two-sample MR study design employed in this analysis is depicted in Figure S1, Supplementary Information..”
		b)	Report any additional statistics (e.g., assessments of heterogeneity across genetic variants, such as I^2, Q statistic or E-value)	Page 13	“To ensure the reliability of the MR results, horizontal pleiotropy and heterogeneity were examined through MR-Egger intercept analysis (*P* > 0.05 indicates no horizontal pleiotropy) and Cochran's Q test, respectively. Considering the potential chance to increase the overall type I

					error during multiple comparisons, we implemented the FDRcorrection...”
13	**Sensitivity analyses and additional analyses**				
		a)	Report any sensitivity analyses to assess the robustness of the main results to violations of the assumptions	Page 4 and supplementary information	Heterogeneity and horizontal pleiotropy were described in table S2
		b)	Report results from other sensitivity analyses or additional analyses	Page 4, Figure S2	“These associations were robust to heterogeneity and pleiotropy, with leave-one-out sen-sitivity analysis showing no substantial changes (Figure S2) and null causation in reverse MR (Supplementary Tables S2, S3). ....”
		c)	Report any assessment of direction of causal relationship (e.g., bidirectional MR)	Page 5	“2.3 Bidirectional interactions between gut microbiota and the genetic factors of ASD..”
		d)	When relevant, report and compare with estimates from non-MR analyses	Page 4-5	See in part 1 results “2.1 Dietary preferences and quality score among individuals with and without ASD”
		e)	Consider additional plots to visualize results (e.g., leave-one-out analyses)	Figure S2, Page 4	Supplementary Figure S2
	**DISCUSSION**				
14	**Key results**		Summarize key results with reference to study objectives	Page 8	“In this study, consistent with observational findings,... The gut microbiome exhibited bi-directional roles in ASD. Among the 13 microbial taxa genetically associated with ASD, *Clostridia*, *Turicibacter*, *Lachnospiraceae*, and *Streptococcus* were further explored as significant mediators of the associations between genetic predisposition to ASD and less healthy dietary preferences.”
15	**Limitations**		Discuss limitations of the study, taking into account the validity of the IV assumptions, other sources of potential bias, and imprecision. Discuss both direction and magnitude of any potential bias and any efforts to address them	Page 9-10	“However, several research limitations should be noted. ...” to the end of this paragraph.
16	**Interpretation**				
		a)	Meaning: Give a cautious overall interpretation of results in the context of their limitations and in comparison with other studies	Page 10	The last paragraph in the discussion, cautious overall interpretations were made.

		b) Mechanism: Discuss underlying biological mechanisms that could drive a potential causal relationship between the investigated exposure and the outcome, and whether the gene-environment equivalence assumption is reasonable. Use causal language carefully, clarifying that IV estimates may provide causal effects only under certain assumptions	Page 9	“Moreover, certain microbial taxa can influence appetite and food preferences.” and the following two paragraphs.
		c) Clinical relevance: Discuss whether the results have clinical or public policy relevance, and to what extent they inform effect sizes of possible interventions	Page 9-10, Page 5	“suggesting its depletion should be the upstream of dietary intake rather than the consequence of diet.”, “These cues require us to further explore how gut microbiota products regulate dietary preferences through immune response in ASD”, and our study provides novel insight into the direction of diagnosed ASD and certain undesired dietary preferences in adulthood, highlighting the mediation effects of the gut microbiome”, and “decreased GLP-1 stimulated insulin secretion with the effect size of -3.010 [-5.380, -0.640] (FDR = 0.030) in the MR Egger result (Supplementary Table S2 and S3). These findings suggest that individuals with ASD may have altered metabolic responses that influence their dietary preferences and appetite regulation.”
17	**Generalizability**	Discuss the generalizability of the study results (a) to other populations, (b) across other exposure periods/timings, and (c) across other levels of exposure	Page 9-10	“Finally, despite the large scale of the UK Biobank, the limited size of the ASD cohort presents a risk of selection bias and the reliance on European-ancestry GWAS may not fully represent broader populations. Although we conducted ....delve deeper into the gut microbiome-mediated mechanism. ”
	**OTHER INFORMATION**			
18	**Funding**	Describe sources of funding and the role of funders in the present study and, if applicable, sources of funding for the databases and original study or studies on which the present study is based	Page 15	Acknowledgements
19	**Data and data sharing**	Provide the data used to perform all analyses or report where and how the data can be accessed, and reference these sources in the article. Provide the statistical code needed to reproduce the results in the article, or report whether the code is publicly accessible and if so, where	Page 14	Data sources were described.
20	**Conflicts of Interest**	All authors should declare all potential conflicts of interest	Page 15	Have stated

This checklist is copyrighted by the Equator Network under the Creative Commons Attribution 3.0 Unported (CC BY 3.0) license.

1. Skrivankova VW, Richmond RC, Woolf BAR, Yarmolinsky J, Davies NM, Swanson SA, et al. Strengthening the Reporting of Observational Studies in Epidemiology using Mendelian Randomization (STROBE-MR) Statement. JAMA. 2021;under review.

2. Skrivankova VW, Richmond RC, Woolf BAR, Davies NM, Swanson SA, VanderWeele TJ, et al. Strengthening the Reporting of Observational Studies in Epidemiology using Mendelian Randomisation (STROBE-MR): Explanation and Elaboration. BMJ. 2021;375:n2233.
